# Three-dimensional printing versus conventional machining in the creation of a meatal urethral dilator: development and mechanical testing

**DOI:** 10.1186/s12938-020-00799-8

**Published:** 2020-07-01

**Authors:** Michael Y. Chen, Jacob Skewes, Ryan Daley, Maria A. Woodruff, Nicholas J. Rukin

**Affiliations:** 1 Science and Engineering Faculty, Queensland University of Technology, Brisbane, Australia; 2 Department of Urology, Redcliffe Hospital, Anzac Ave, Redcliffe, QLD 4020 Australia; 3 School of Medicine, University of Queensland, Brisbane, QLD Australia; 4 Herston Biofabrication Institute, Metro North Hospital and Health Service, Brisbane, Australia

**Keywords:** Three-dimensional printing, Urology, Urethral stricture, Intermittent urethral catheterisation

## Abstract

**Background:**

Three-dimensional (3D) printing is a promising technology, but the limitations are often poorly understood. We compare different 3D printing methods with conventional machining techniques in manufacturing meatal urethral dilators which were recently removed from the Australian market.

**Methods:**

A prototype dilator was 3D printed vertically orientated on a low-cost fused deposition modelling (FDM) 3D printer in polylactic acid (PLA) and acrylonitrile butadiene styrene (ABS). It was also 3D printed horizontally orientated in ABS on a high-end FDM 3D printer with soluble support material, as well as on an SLS 3D printer in medical nylon. The dilator was also machined in stainless steel using a lathe. All dilators were tested mechanically in a custom rig by hanging calibrated weights from the handle until the dilator snapped.

**Results:**

The horizontally printed ABS dilator experienced failure at a greater load than the vertically printed PLA and ABS dilators, respectively (503 g vs 283 g vs 163 g, *p* < 0.001). The SLS nylon dilator and machined steel dilator did not fail. The steel dilator is the most expensive with a quantity of five at 98 USD each, but this decreases to 30 USD each for a quantity of 1000. In contrast, the cost for the SLS dilator is 33 USD each for five and 27 USD each for 1000.

**Conclusions:**

Low-cost FDM 3D printing is not a replacement for conventional manufacturing. 3D printing is best used for patient-specific parts, prototyping or manufacturing complex parts that have additional functionality that cannot otherwise be achieved.

## Background

Three-dimensional (3D) printing, or additive manufacturing, is a promising technology that can create complex 3D structures layer by layer and is increasingly being used in biomedical and urological research to create patient-specific and geometrically complex constructs in an economical way [1, 2]. There are a wide variety of 3D printing techniques now available. Fused deposition modelling (FDM) printing is the most commonly used and lowest cost approach since the expiry of patents. In describing the low cost of 3D printing, many medical research papers quote a price of USD 300 for a 3D printer [3–5]. However, many clinicians may be unaware of the quality of print that can be achieved with such a low-cost 3D printing method when compared to conventional manufacturing. In our experience, to achieve a consistent result with low-cost FDM printing a printer cost of 1000 to 3000 USD is more realistic.

Instead of FDM, research groups frequently employ more advanced 3D printing methods such as stereolithography (SLA) or selective laser sintering (SLS). For example, in the field of urology, many groups have explored the use of 3D-printed prostate and kidney models using SLA technology [6–8]. Many non-invasive uses for 3D printing in urology have been explored ranging from surgical simulation [9–12], histopathological correlation [13, 14], augmented reality surgery [15, 16] and anatomical models [6, 7, 17–20]. However, the next frontier in 3D printing research is the development of clinically useful 3D printed equipment, tools and implants. To that end, some have begun exploring whether 3D printing could be used to create basic surgical equipment. This includes equipment such as forceps [21] and retractors [22] which are currently mass manufactured using conventional methods. Whilst interesting, this raises the question of whether 3D printing these basic devices will be cost-effective or clinically safe.

However, many clinicians are unaware of the limitations of low-cost FDM 3D printing and the strengths of conventional manufacturing techniques.

Whilst FDM printing can be low cost and accessible to hospitals around the world, the quality of the parts produced may not be mechanically consistent enough, particularly in healthcare where standards of safety are high. To produce higher quality parts, the setup cost of a 3D printer or machine will be higher.

In the urological field, Park et al. [23] used 3D printing to create a ureteric stent prototype which prevented reflux in vitro. This is a valid use of 3D printing to prototype a part in small quantities. 3D-printed ureteric stents and laparoscopic trochars also showed feasibility in porcine models [24].

3D printing in medicine has perhaps advanced the most in the field of orthopaedic surgery. 3D-printed acetabular cups demonstrate the strengths of 3D printing as they can be patient-specific, but also are highly porous to encourage bone ingrowth which conventional manufacturing cannot achieve [25], however the long-term outcomes remain to be seen.

Meatal stenosis is an abnormal narrowing of the urethral meatus which is the distal opening of the urethra. It can arise secondary to skin disorders or instrumentation injury. For example the incidence of meatal stenosis following circumcision is around 7–11% [26, 27]. Self-dilatation by patients at home is often used as a treatment option in the initial stages with surgery reserved for persisting issues [28, 29].

The Cook© meatal dilator we used at our institutions was recently removed from the Australian market and is no longer available. This prompted us to explore the production of a new meatal dilator which we could potentially 3D print in-house at our institution. Meatal dilators are used by patients at home and do not have to be sterile, thus being a simple low-risk device with which to compare manufacturing methods [29]. In this study, we compare cost and mechanical strength of dilators made with four approaches: (i) lowest cost FDM 3D printing of a vertically orientated dilator; (ii) horizontally orientated dilator on a higher quality FDM 3D printer; (iii) the more advanced 3D printing technique of SLS and (iv) conventional lathe machining.

## Results

### Dilator creation

**Fig. 1:**
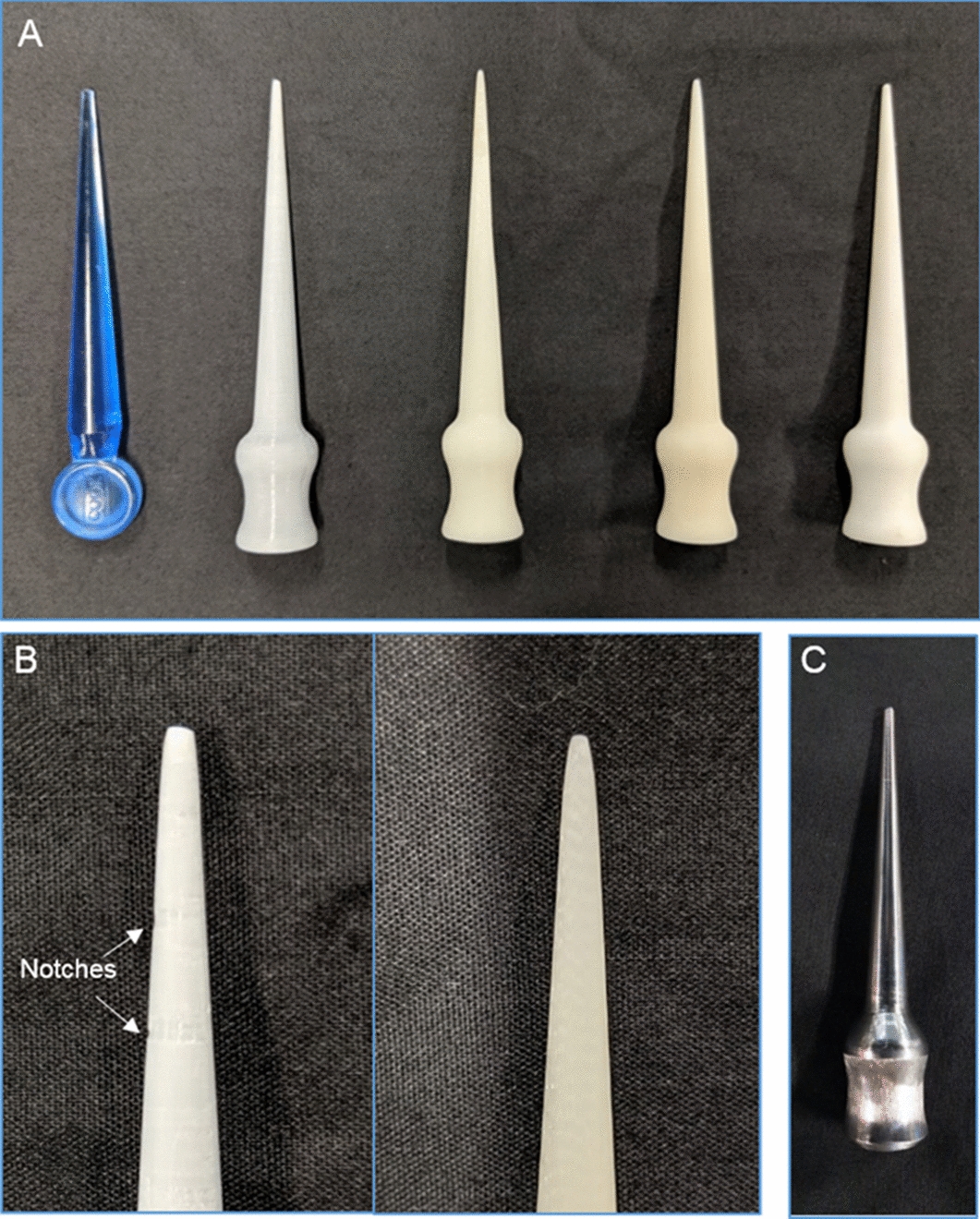
**a** 3D-printed meatal urethral dilators (left to right): commercial Cook© meatal dilator, vertical PLA, vertical ABS, horizontal ABS, SLS nylon. **b** Comparison in the 3D print quality of the tips of the vertically orientated ABS dilator (left) which shows notches on the left where imperfections have developed and horizontally orientated ABS dilator (right) which was printed with soluble support material and has a smoother result. **c** Machined stainless steel dilator

Three dilator prototypes were created using each different method. One issue encountered during the FDM printing process of the vertically orientated dilators was that each layer at the tip was so small that there was insufficient time for the material to cool after extrusion. In addition, the tiny layers led to some instability of the part which meant the fine details at the tip were not accurately printed (Fig. 1). This defect occurred in all three dilator parts for vertical PLA and ABS, despite attempts to manually increase the time between layers such as by slowing the print speed. In contrast, the horizontally printed ABS dilator was able to be printed smoothly with the addition of dual extrusion soluble support material.

The SLS nylon dilator printed without issue. All 3D printed dilators were smoothed with sandpaper. The machined steel dilator was manufactured without issue and polished to smooth the surface (Fig. 4).

### Mechanical testing

All three FDM-printed prototype meatal dilators snapped cleanly during testing (Table 1). As expected, the horizontally printed ABS dilator was more ductile than the vertically printed ABS and PLA dilators, respectively (503 g vs 283 g vs 163 g, *p* < 0.001). Figure 5 shows just before breaking how the ductility of the horizontally printed ABS dilator allows it to bend to a far greater degree.

**Table 1:** Comparison of mechanical testing results between dilator prototypes

Prototype	Failure angle, degrees	Maximum weight test number 1 (g)	Maximum weight test number 2 (g)	Maximum weight test number 3 (g)	Maximum weight in g, mean (SD)
Vertical ABS	12	300	270	280	283 (15)
Vertical PLA	10	150	220	120	163 (51)
Horizontal ABS	40	500	510	500	503 (6)
SLS nylon	No failure	> 5000	Not repeated	Not repeated	> 5000
Machined stainless steel	No failure	> 10,000	Not repeated	Not repeated	> 10,000

**Fig. 2:**
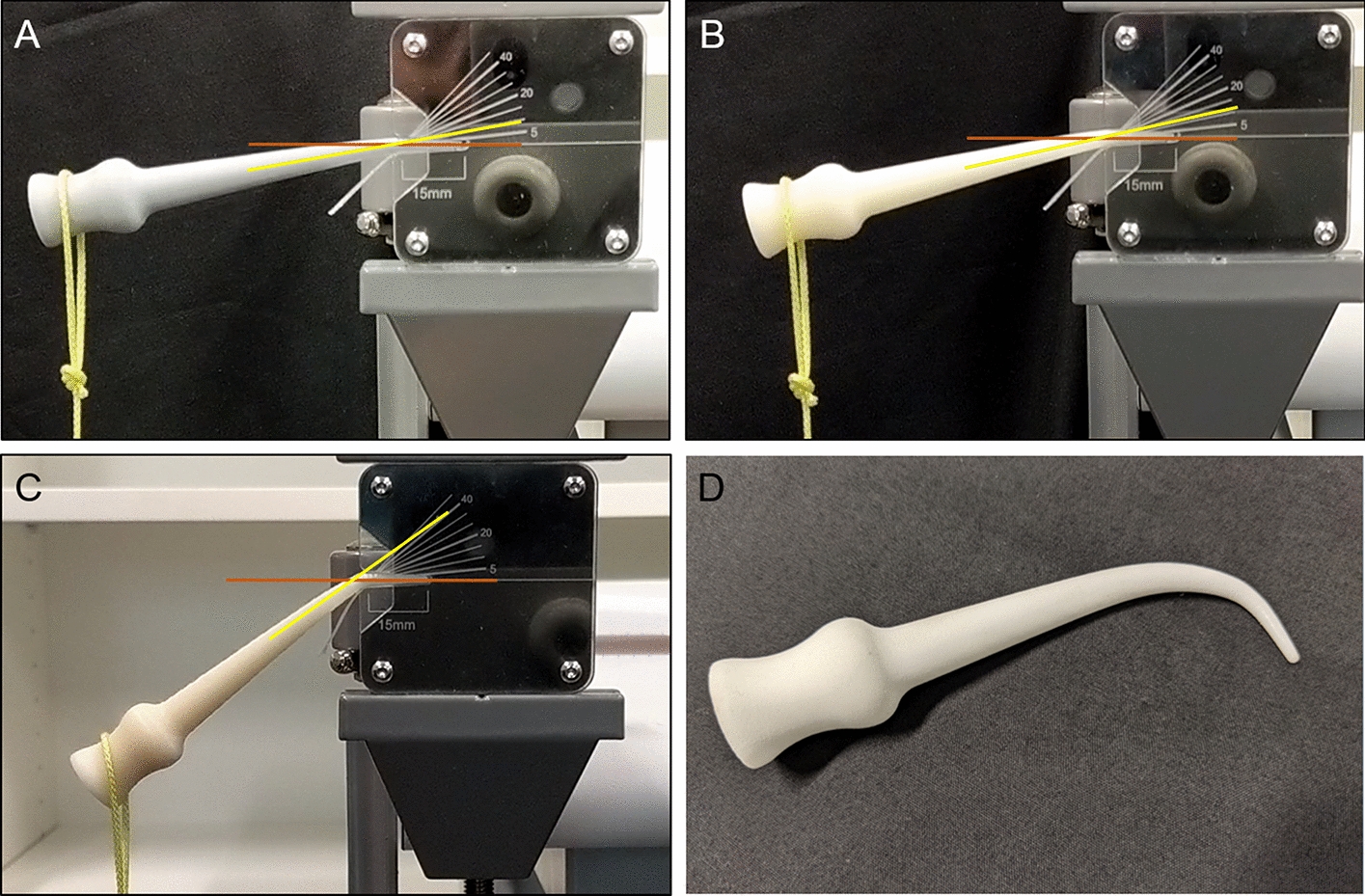
A freeze frame of the video the moment before the vertical PLA (**a**), vertical ABS (**b**) and horizontal ABS (**c**) dilators snapped. The approximate angle of bending before snapping was estimated as 10°, 12° and 40°, respectively. SLS nylon 3D-printed dilator (**d**) after applying approximately 5 kg of manual pressure to the tip bent but did not break

The SLS nylon dilator was unexpectedly strong and elastic and did not fail during our mechanical testing with calibrated weights. When the weight reached around 1000 g, the dilator elastically deformed and slipped out of the rig. When approximately 5000 g of manual force was directly applied to the nylon dilator tip, it did not snap but began to bend (Fig. 2). As expected, the steel dilator was not able to be bent or snapped even when approximately 10,000 g was manually to the tip.

### Cost analysis

Table 2 summarises the comparison between the dilator types in terms of various costs. Assuming an order quantity of five from an external company, conventional machining is more costly than 3D printing with a unit price of USD 124. However, if we assume a quantity of 1000 then the unit price of the stainless steel dilators drops to around USD 18. 3D printing does not have the same benefit in producing large quantities and the unit price plateaus at a lower quantity. For example, the SLS nylon dilators cost USD 35 each for a quantity of five, but USD 27 each for a quantity of 100 which then plateaus to 16 for 1000 parts.

**Table 2:** Comparison between different meatal urethral dilators in cost

Prototype	Material cost ~ USD	Printer/machine	Printer/machine cost, ~ USD	Total labour time, h	Cost per dilator (assuming order quantity of 5)	Cost per dilator (assuming order quantity of 1000)
Vertical ABS	1	Ultimaker 2+	2500	3	12	4
Vertical PLA	1	Ultimaker 2+	2500	3	11	3
Horizontal ABS	6	Fortus 400	185,000	4	45	25
SLS nylon	3	Formiga P100	175,000	1.5	35	16
Machined stainless steel	4	HAFCO metal master 320-G	2400	0.75	98	18

Injection moulding allows for very low cost for mass manufacturing of devices, but has a high setup tooling cost which makes it unsuitable for low quantities and therefore was not used in this prototyping study [30]. However, quotes from external companies report a tooling cost of around USD 4500 to setup the process. Following this, a variety of thermoplastic materials can be used to manufacture parts at very low cost. For example, the cost per part in ABS with injection moulding is around USD 0.50.

## Discussion

In this study we have compared different materials as well as different manufacturing methods in the creation of meatal urethral dilator prototypes. Low-cost FDM-printed parts were mechanically weak and therefore potentially unsafe for clinical use. We have performed relatively simple mechanical testing which would be suitable for a low-risk device such as a meatal urethral dilator. However, higher risk devices that are required to be sterile would need more rigorous testing. Unfortunately, as commercial meatal dilators were taken off the Australian market, we were unable to perform mechanical testing on commercially available products. In this preliminary analysis, we have tested only a small selection of materials and manufacturing techniques which we felt most appropriate for this purpose.

Our preliminary cost analysis has shown that SLS printing of meatal dilators could be an efficient alternative to machining. Although the cost of USD 175,000 for the SLS printer used in this study may present a cost barrier for many institutions, with the expiry of patents the cost of SLS printers continues to decrease over time [31]. Any discussion around cost is likely to change in the future as 3D printing technology continues to evolve and become cheaper and more accessible. Our quoted prices are from single commercial sources which should be taken as approximations only, valid at the present time (2019). Even during the time of around 1 year of this project many of these prices changed significantly which makes effective cost analysis difficult.

One of the difficulties in designing this study was the lack of data on what threshold of mechanical strength would be needed. Although the use of intermittent self-catheterisation to prevent stricture recurrence is established in the literature [32], most studies are focused on dilatation using long urethral catheters rather than the short meatal dilator in our study. In fact, the literature on meatal dilators is extremely limited [29]. Therefore, our design aims to stress the dilator at its weakest point, although this may not occur in clinical practice. Based on clinical experience, we are confident that the SLS dilator not breaking even at 5000 g of force is sufficiently safe for clinical use. Even the horizontal ABS dilator which could take 500 g of weight and only failed at a 40 degree bend would likely be safe as a patient is unlikely to bend the dilator to that angle during use in the urethra. If a portion of the dilator did snap it would need to be retrieved via flexible cystoscopy by a urologist under local anaesthetic with overall minimal morbidity to the patient.

In contrast with previous studies in this area in urology, this project did not aim to innovate with the addition of 3D printing. Park et al. [23] demonstrated how 3D printing can be used to prototype novel ureteric stents that do not reflux which could potentially reduce the discomfort patients experience from the stents. However, if such a prototype entered mainstream use then 3D printing would not necessarily be the best method to continue production. Del Junco et al. [24] tested 3D-printed ureteric stents and laparoscopic trochars in porcine models and concluded it was feasible, despite the initial functional failures they described and with no discussion on cost.

The strengths of 3D printing are in the creation of patient-specific parts, manufacturing complex parts which conventional methods cannot achieve, or prototyping. Despite the enormous potential of the technology, 3D printing should not be viewed yet as a replacement for conventional manufacturing techniques. This is especially true for parts needed in high quantities with simple geometry such as the dilator. For example, the quoted unit price of five machined steel dilators is around USD 124, but the unit price of ordering a quantity of 1000 becomes around USD 18. It is also worth noting that with modern machining parts with simple geometry such as the dilator can also be made to be patient-specific, particularly with the aid of computer numerical controlled (CNC) machining [33].

Our study is one of the first to examine the practicalities of using 3D printing techniques to produce low-risk clinically applicable medical devices such as the urethral meatal dilator. Whilst many clinicians may be aware of how low-cost 3D printing can be, we hope to show that there are limitations to low-cost FDM printing. It may be difficult for individual institutions to be able to use low-cost FDM 3D printing to manufacture devices in-house at a quality that is clinically safe and reliable. 3D printing medical research should not seek to replace all conventional techniques in producing simple devices, but instead capitalise on the advantages of 3D printing in creating complex geometries or patient-specific parts. To our knowledge, this is also the first project to investigate a method for mechanically testing the strength of urethral dilators.

## Conclusions

3D printing is not a replacement for all conventional manufacturing techniques at this time. Although low-cost FDM printing technology is easily accessible, the meatal urethral dilators created were mechanically weak and their quality was too inconsistent for clinical use. Clinicians should consider higher quality 3D printing options such as SLS for creating reliable parts which can compete with conventional manufacturing in lower quantities. 3D printing in medicine is best used for patient-specific parts, prototyping, or complex parts that add functionality that cannot be achieved with conventional manufacturing.

## Methods

### Prototype design

**Fig. 3:**
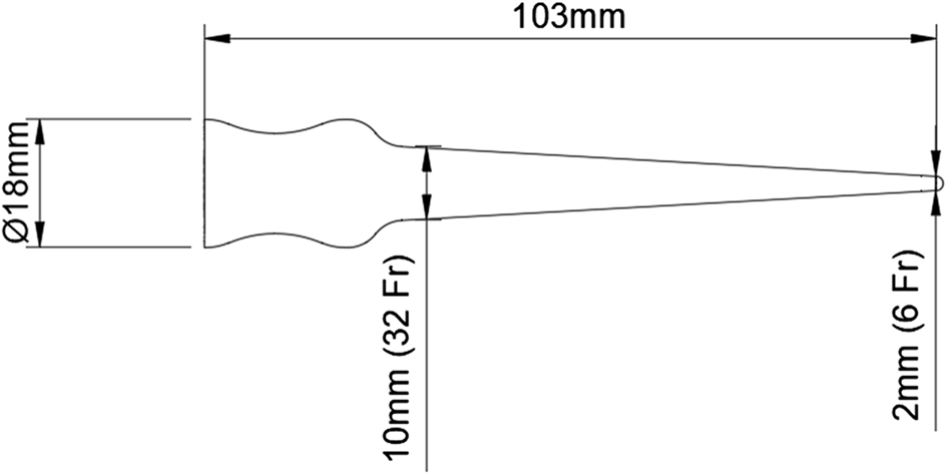
Prototype dilator design and dimensions in millimetres (mm) and French (Fr)

A prototype meatal dilator was created using computer-aided design (CAD) on software Fusion 360 (Autodesk, San Rafael, US). The dilator, excluding the handle, is 90.0 mm in length with a diameter starting from 2.0 mm (6 French) at the tip and 10.7 mm (32 French) at the base (Fig. 3). A handle was added which was designed to be curved to help facilitate machining. This design process took approximately 1 h. The design was done in collaboration with an experienced urethral surgeon who approved the shape, diameter and length.

### Fused deposition modelling

**Fig. 4:**
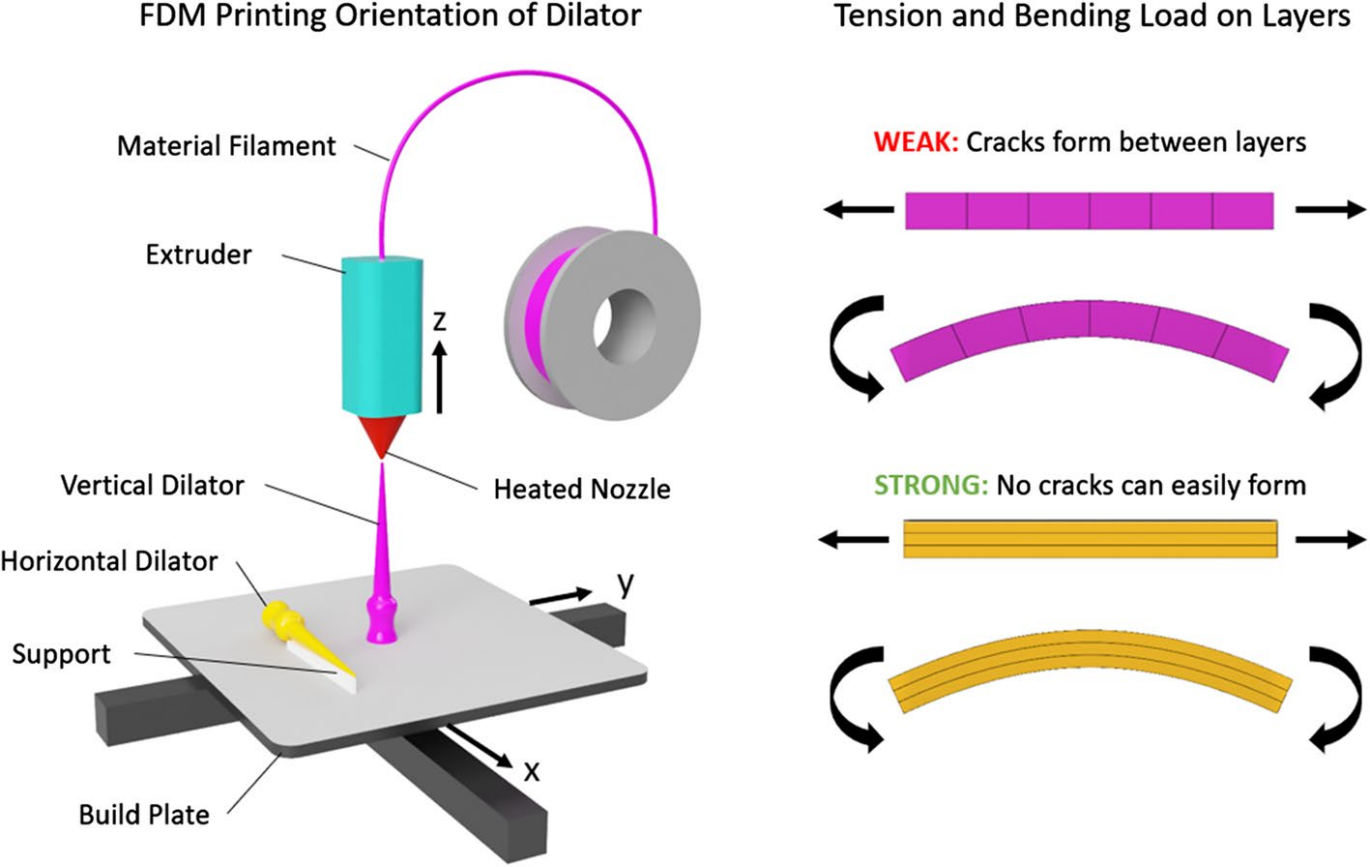
A comparison of how the orientation of the dilator in an FDM 3D printer can affect the mechanical strength at the tip. The vertically orientated 3D-printed dilator (purple) will be more likely to snap completely due to the weakness between the layers compared to the horizontally printed dilator (yellow). However, the downside is that overhanging parts like the tip of the dilator when orientated horizontally requires printing of a support material

FDM extrudes thermoplastic material via a heated nozzle layer by layer and thus it requires printing of support material for any overhanging areas. Therefore, the easiest way to 3D print the dilator prototype without supports is to print it orientated vertically. However, the orientation of an FDM print affects the mechanical properties as the part will be weakest between each layer (Fig. 4). Therefore, a horizontally orientated dilator would be less likely to snap, but would require a more complex and costly 3D printing process.

The prototype dilator was initially 3D printed with a low-cost FDM technique using polylactic acid (PLA) and acrylonitrile butadiene styrene (ABS) orientated vertically. PLA and ABS are the two most common materials used in 3D printing [34]. As we found ABS to be more ductile than PLA as expected, we then printed in ABS orientated horizontally on a higher quality FDM printer using soluble support material. The vertical PLA and ABS dilators were printed on an Ultimaker 2 + (Ultimaker B.V., Geldermalsen, Netherlands) while the horizontal ABS dilators were printed on the Fortus 400 (Stratasys, Eden Prairie, US) which allows dual extrusion of the ABS filament and also a soluble support filament that can be removed after the print is completed.

### Selective laser sintering

An SLS technique was used to 3D print in nylon (polyaurinlactam 2200). This material is approved for medical use and bio-compatible according to EN ISO 10993-1 and USP/level VI/121 °C standards. The SLS technique uses a laser to fuse powder particles layer by layer which helps create a part with more isotropic mechanical properties, with less weakness between layers found in FDM printing. In addition, highly complex parts can be printed without supports. The SLS 3D printer used was the Formiga P100 (EOS, Munich, Germany).

### Conventional machining

The dilator was also manufactured using a manual lathing process in medical grade 316L stainless steel on a HAFCO Metal Master 350 lathe (Hare & Forbes, Sydney, Australia). This process was chosen because the geometry of the dilator is symmetrical and revolute around its central axis.

Using a lathe to reshape a bar of steel into the desired shape, a material volume wastage of approximately 60% can be expected, as opposed to a possible 0% when compared to 3D printing.

### Mechanical testing

**Fig. 5:**
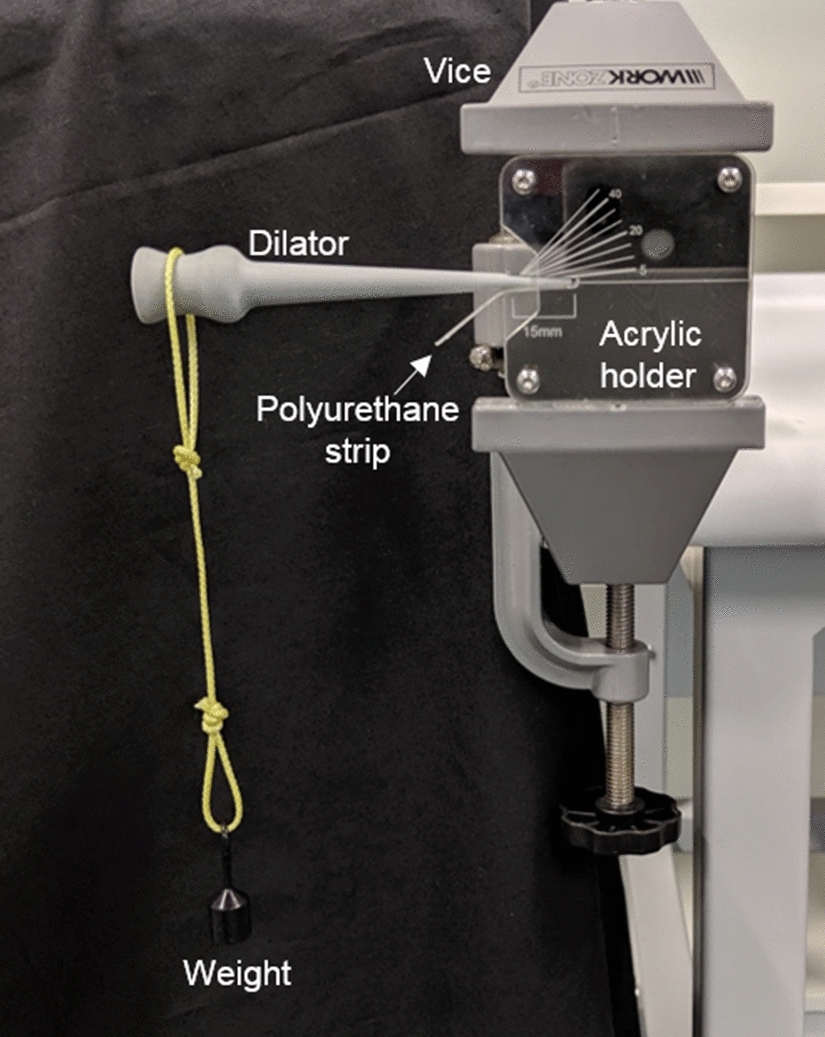
Setup for mechanical testing of dilators. Dilator inserted into custom laser-cut acrylic with markings for tip length and angles and held in place by vice. Calibrated weights are hung from handle of dilator

To test the mechanical strength of the dilators, we created a “worst-case scenario” in which all the mechanical stress was concentrated at the tip where it is the weakest. A custom rig was constructed using a laser-cut acrylic to hold the dilator (Fig. 5). The acrylic has markings to display the bending angle of the dilator. A strip of polyurethane is placed at the bottom of the dilator tip to provide a softer surface, more similar to urethra. Calibrated test weights were then hung from the handle end of the dilator in increasing amounts. The process was video recorded to estimate the breaking angle and weight of the dilators. Three dilators were created in each method and all three were tested in the same way.

### Analysis

The primary outcomes were the cost of the dilators and their mechanical strength. The cost analysis of the dilators and 3D printers was based on prices quoted by 3D printing distribution companies. Cost per dilator was based on an assumption of an institution ordering a quantity of five dilators from a company offering 3D printing or machining. Mean and standard deviation breaking weight were calculated for the three tests on each dilator type. One-way ANOVA was used to determine statistical significance using Stata (StataCorp. 2017. Stata Statistical Software: Release 15. College Station, TX: StataCorp LLC).

## Data Availability

Data sharing is not applicable to this article as no datasets were generated or analysed during the current study.

## Abbreviations

3D printing: Three-dimensional printing
FDM: Fused deposition modelling
SLA: Stereolithography
SLS: Selective laser sintering
ABS: Acrylonitrile butadiene styrene
PLA: Polylactic acid
CAD: Computer-aided design
